# Speeding up the detection of non-iconic and iconic gestures (SPUDNIG): A toolkit for the automatic detection of hand movements and gestures in video data

**DOI:** 10.3758/s13428-020-01350-2

**Published:** 2020-01-23

**Authors:** Jordy Ripperda, Linda Drijvers, Judith Holler

**Affiliations:** 1 Donders Institute for Brain, Cognition, and Behaviour, Radboud University, Montessorilaan 3, 6525 HR Nijmegen, The Netherlands; 2 Max Planck Institute for Psycholinguistics, Wundtlaan 1, 6525 XD Nijmegen, The Netherlands

**Keywords:** Automatic movement detection, Gesture, Openpose, Annotation, Hand, Methodology, Motion tracking

## Abstract

In human face-to-face communication, speech is frequently accompanied by visual signals, especially communicative hand gestures. Analyzing these visual signals requires detailed manual annotation of video data, which is often a labor-intensive and time-consuming process. To facilitate this process, we here present SPUDNIG (SPeeding Up the Detection of Non-iconic and Iconic Gestures), a tool to automatize the detection and annotation of hand movements in video data. We provide a detailed description of how SPUDNIG detects hand movement initiation and termination, as well as open-source code and a short tutorial on an easy-to-use graphical user interface (GUI) of our tool. We then provide a proof-of-principle and validation of our method by comparing SPUDNIG’s output to manual annotations of gestures by a human coder. While the tool does not entirely eliminate the need of a human coder (e.g., for false positives detection), our results demonstrate that SPUDNIG can detect both iconic and non-iconic gestures with very high accuracy, and could successfully detect all iconic gestures in our validation dataset. Importantly, SPUDNIG’s output can directly be imported into commonly used annotation tools such as ELAN and ANVIL. We therefore believe that SPUDNIG will be highly relevant for researchers studying multimodal communication due to its annotations significantly accelerating the analysis of large video corpora.

## Introduction

Spoken language is mostly used in multimodal, face-to-face contexts. In addition to speech, face-to-face communication involves a plethora of visual articulators, such as manual gestures. Due to their close relation to speech, manual gestures (henceforth: gestures) have often been the focus of multimodal research in the domains of psychology, anthropology, linguistics and neuroscience (Goldin-Meadow, 2005; Kendon, 2004; McNeill, 1992). For example, gestures can be used to refer to objects, events, locations and ideas, and can convey semantic information integral to a speaker’s message (Holler & Beattie, 2003; Holler & Wilkin, 2009; Hostetter, 2011; McNeill, 1992). Previous work has demonstrated that this information is integrated with the speech signal, processed by the listener, and that it facilitates language comprehension (Drijvers & Özyürek, 2017; Holler, Shovelton, & Beattie, 2009; Kelly, Barr, Church, & Lynch, 1999; Kelly, Kravitz, & Hopkins, 2004; Kelly et al., 2010; Ozyurek, 2014).

One of the main challenges for studies on human communication in face-to-face contexts is the labor-intensive and time-consuming manual annotation of the occurrence and timing of such gestures. These manual analyses are often performed on the basis of pre-defined coding schemes (e.g., Dael, Mortillaro, & Scherer, 2012; McNeill, 1992; Zhao & Badler, 2001), and using annotation tools such as ANVIL (Kipp, 2001) or ELAN (Wittenburg, Brugman, Russel, Klassmann & Sloetjes, 2006). These annotation tools log manually made annotation entries time-stamped in relation to the video – an extremely useful step – but they do not speed up or automatize the annotation process itself. Gestures still need to be detected based on the human eye and their begin and end points need to be entered manually. Moreover, annotators need to be trained to perform this procedure, and multiple coders need to be involved to establish inter-rater reliability. This results in an extremely time-consuming process even for individual experiments, and especially so for more large-scale, corpora-based research projects. Facilitating the gesture annotation process through the development of automated techniques thus would advance multimodal communication research significantly.

Recent technological advances have opened up exciting possibilities for the automatic analyses of movement parameters (such as space, motion trajectories, size, distance, and velocity). These automatic analyses are often performed on the basis of device-based optic marker or markerless motion tracking systems, such as Polhemus Liberty (Vermont, USA; http://polhemus.com; Liberty Latus Brochure, 2012), Optotrak (Northern Digital, Waterloo, Canada), Microsoft Kinect (Zhang, 2012), and Leap Motion (San Francisco, USA; http://leapmotion.com).

Alternatively, when motion capture systems are unavailable, video-based tracking systems can be used to automatically track movements. These video-based analysis methods include pixel differentiation methods (e.g., Paxton & Dale, 2013), computer-vision methods relying on deep learning, (e.g., OpenPose; Cao, Hidalgo, Simon, Wei, & Sheikh, 2018; Cao, Simon, Wei, & Sheikh, 2016) and Deeplabcut (Mathis et al., 2018); for an overview and discussion of the different methods see Pouw, Trujillo, & Dixon, 2018). Recent work has validated that video-based tracking systems perform equally well in estimating gesture characteristics (such as movement peaks) as device-based motion tracking systems (Pouw et al., 2018).

The decision on what type of motion tracking method is most suitable for a user’s research question is often dependent on the user’s access to such methods. Access can be limited by the cost level of motion capture systems or specific computer-based hardware requirements (e.g., access to a graphics processing unit (GPU), but also due to the requirement of technical expertise to apply existent video-based motion tracking methods (first and foremost, expertise in specific programming languages). Moreover, existing toolkits on (semi-)automatic gesture and movement analyses still require, as a first step, the *manual identification* of the gesture/movement events that need to be analyzed (e.g., Pouw et al., 2018; Trujillo, Vaitonyte, Simanova, & Özyürek, 2019), require motion capture data as input for detection of gesture initiation and termination (e.g., by using EPAA, see Hassemer, 2015), or are not suited to track fine-grained finger movements (e.g., Beugher, Brône, & Goedemé, 2018).

To overcome these limitations, we here present SPUDNIG (**SP**eeding **U**p the **D**etection of **N**on-iconic and **I**conic **G**estures), a newly developed open-source toolkit including an easy-to-use graphical user interface (GUI) for the automatic detection of hand movements and gestures. Similar to De Beugher et al., (2018), we define ‘detection’ as segmenting movement sequences from non-movement sequences (note that *recognition* would involve distinguishing which of these movements are gestures and which of these movements are not gestures). SPUDNIG uses OpenPose as input for continuous, video-based motion tracking of movements and gestures, and subsequently uses observed changes in x/y coordinates of user-defined key points in the body to automatically detect movement initiation and termination. SPUDNIG does not require the use of motion capture hardware devices, nor does it require in-depth technical knowledge to adapt parameters used for gesture detection, or expertise in programming languages. In what follows, we provide a proof-of-principle and validation of our “off-the-shelf” gesture detection method by comparing the output of SPUDNIG to manually annotated gesture data in the ELAN annotation tool. We will test its performance for both non-iconic and iconic gestures. Please note that SPUDNIG, in its current form, was not designed to entirely eliminate the need of a human coder. Its current version is not able to *recognize* gestural from non-gestural movement, but is able to *detect* movement initiation and termination. Our aim here is to evaluate its overlap with gestures identified by a trained human coder, and to provide a toolkit that significantly reduces the need of a human coder, limiting it to the removal of false positives (i.e., non-gestural movements) at the end of the machine-based annotation process.

## Methods

### SPUDNIG: speeding up the detection of non-iconic and iconic gestures

#### OpenPose

SPUDNIG is partially based on output created by OpenPose (https://github.com/CMU-Perceptual-Computing-Lab/openpose). OpenPose is a video-based motion tracking method that uses computer-vision methods to estimate body parts from 2D frames (e.g., body, face, and hands, see: Cao et al., 2016, 2018). Specifically, it uses convolutional neural networks as a deep learning method to predict the location of body parts and the occurrence of body part motion. This makes OpenPose more robust to background noise than other video-based tracking methods, such as pixel differentiation, and therefore perhaps more suited to the study of human communication in face-to-face contexts. Note that other methods, such as AlphaPose (Fang et al., 2017), have demonstrated to outperform OpenPose at the task of pose estimation. However, AlphaPose can only estimate body pose, and cannot detect key points in the hands. As SPUDNIG requires the detection of fine-grained hand and finger movements, OpenPose was chosen as the basis for SPUDNIG.

#### Movement/no-movement detection

Per frame, OpenPose provides x- and y-coordinates of 25 key points divided over the body, and 21 key points per hand. SPUDNIG then extracts these coordinates per frame and outputs them in three .csv files (body key points / left-hand key points / right-hand key points). Out of these files, it selects a set of eight (default) key points to estimate the initiation and termination of hand movements. The key points that are taken into account from the body are both wrists, and the key point between the wrist and elbow for each arm. Coordinate changes in those points mainly reflect large hand movements. The key points that are taken into account from the left- and right-hand are the tip of the index finger and the tip of the thumb of each hand. The selection of these key points, as well as the number of key points, were the result of a careful piloting phase in which we investigated the trade-off between false positives and false negatives. Adding more key points resulted in more false positives, and removing key points resulted in more false negatives. However, note that the user can edit the number and selection of the key points for their own research purposes to alter this weighting.

**Fig. 1:**
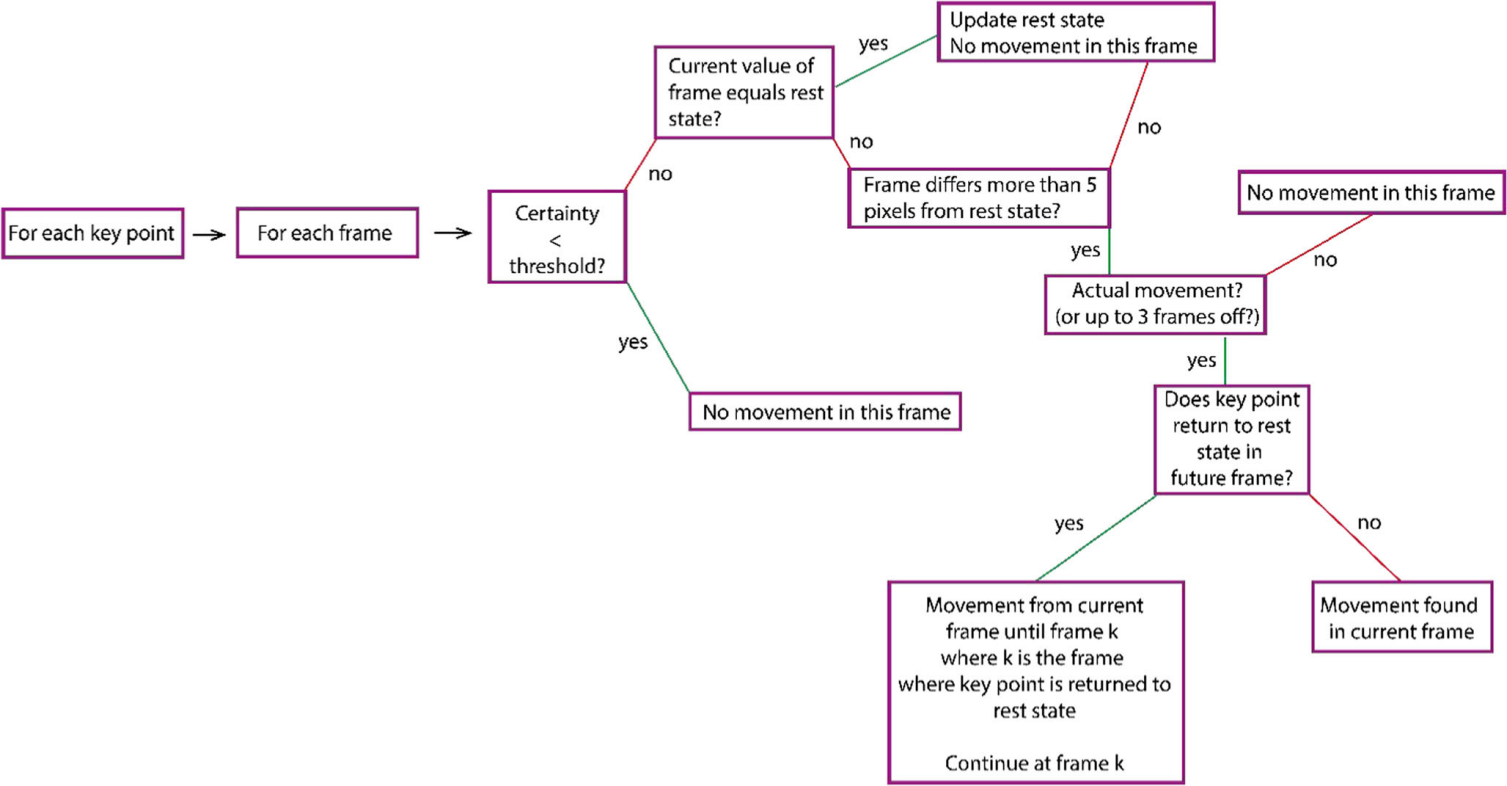
Flowchart of the SPUDNIG algorithm

We have visualized our algorithm in a flow chart in Fig. 1. For each frame, SPUDNIG calculates per key point whether a movement is taking place or not. First, it checks whether the reliability of the OpenPose output is above a certain threshold (default = 0.3, which can be altered by the user). If the reliability of a key point in a frame is below the reliability threshold, the script stops checking for potential movement and indicates that no movement is detected in the respective frame, before continuing to the next frame. If the reliability is above the threshold, the script continues and determines whether the key point in question is part of a rest state or part of a movement.

Rest states (i.e., the hands not being engaged in movement) are determined by checking the x/y-coordinates of a certain manual key point over a span of 15 frames (corresponding to 600 ms when using 25 frames per second (fps), as one frame corresponds to 40 ms (1000 ms/25 frames)), with the current frame (*k*) being the midpoint (i.e., frame *k*-7 until frame *k*+7 are checked). If the x/y-coordinates of these frames differ less than ten pixels from the coordinates of the current frame *k*, with an overall certainty threshold of 0.7 (i.e., 70% of the frames show less than ten pixels difference), SPUDNIG indicates that frame *k* is part of a rest state and that no movement is occurring. It then continues to the next frame. If the certainty threshold of 0.7 is not met, SPUDNIG continues to assess whether frame *k* is part of a movement. It then first checks whether frame *k* differs more than five pixels from the previous frame. If it does, it evaluates whether out of the five frames following frame *k*, minimally three frames differ more than five pixels from frame *k*. This extra check of the 5+ pixel criterion is performed to ensure that a movement is not falsely recognized due to slight shifts in the x/y-coordinates of a certain key point. Again, the particular criterion chosen was based on minimizing false negatives and false positives during our piloting, but can be altered in the code.

If movement is detected, SPUDNIG searches for a rest state in the upcoming 300 frames (~12 s, when using 25 fps), according to the method described above. If a new rest state cannot be found, SPUDNIG repeats this procedure with a slightly larger range in order to increase the chance of finding a rest state.

The above-mentioned process is repeated for all default key points and results in a list that indicates per frame, per key point, whether movement was detected or not. The resulting lists are then merged for all key points to ensure all manual movements are captured, even when reliability for one of the default key points is low. This approach minimized false negatives, and ensured that when one or some of the key points had lower reliability, movements could still be detected.

#### Post-processing

After SPUDNIG has determined which frames contain movements, it removes movements smaller than four frames to diminish the number of false positives. Additionally, movements that occurred within four frames from each other are merged into one to account for small pauses or holds in movements. These settings are based on an extensive trial phase with videos different from the ones we used to validate our toolkit, with the aim to arrive at settings that would keep both the number of false positives and false negatives at a minimum. To most optimally determine the threshold for movement detection, the parameters were set by, as a primary criterion, keeping the number of false negatives as low as possible, combined with a secondary criterion that reduced the number of false positives as much as possible, given criterion one. Note that the user might want to consider changing these settings, depending on their research goal or type of video. These specific parameters can, if needed, easily be altered in the source code.

Based on the fps, the timing of each movement is calculated by converting the frame number to hh:mm:ss.ms format. This is used to create a .csv file containing all start and end times of annotations, which is compatible with commonly used annotation tools, such as ANVIL (Kipp, 2001) and ELAN (Wittenburg et al., 2006).

#### Graphical user interface

SPUDNIG is implemented in an easy-to-use GUI, for which no programming, specific technological knowledge, or GPU hardware is needed. Below, we provide a short tutorial on how to use the SPUDNIG GUI to analyze video data.

#### Brief SPUDNIG tutorial

##### Step 1 – Loading the video file

After starting the application, an .avi video file can be selected for analysis (see Fig. 1). A frame of the selected video file will appear on the screen.

##### Step 2 – Video analysis & selecting analysis parameters

**Fig. 2:**
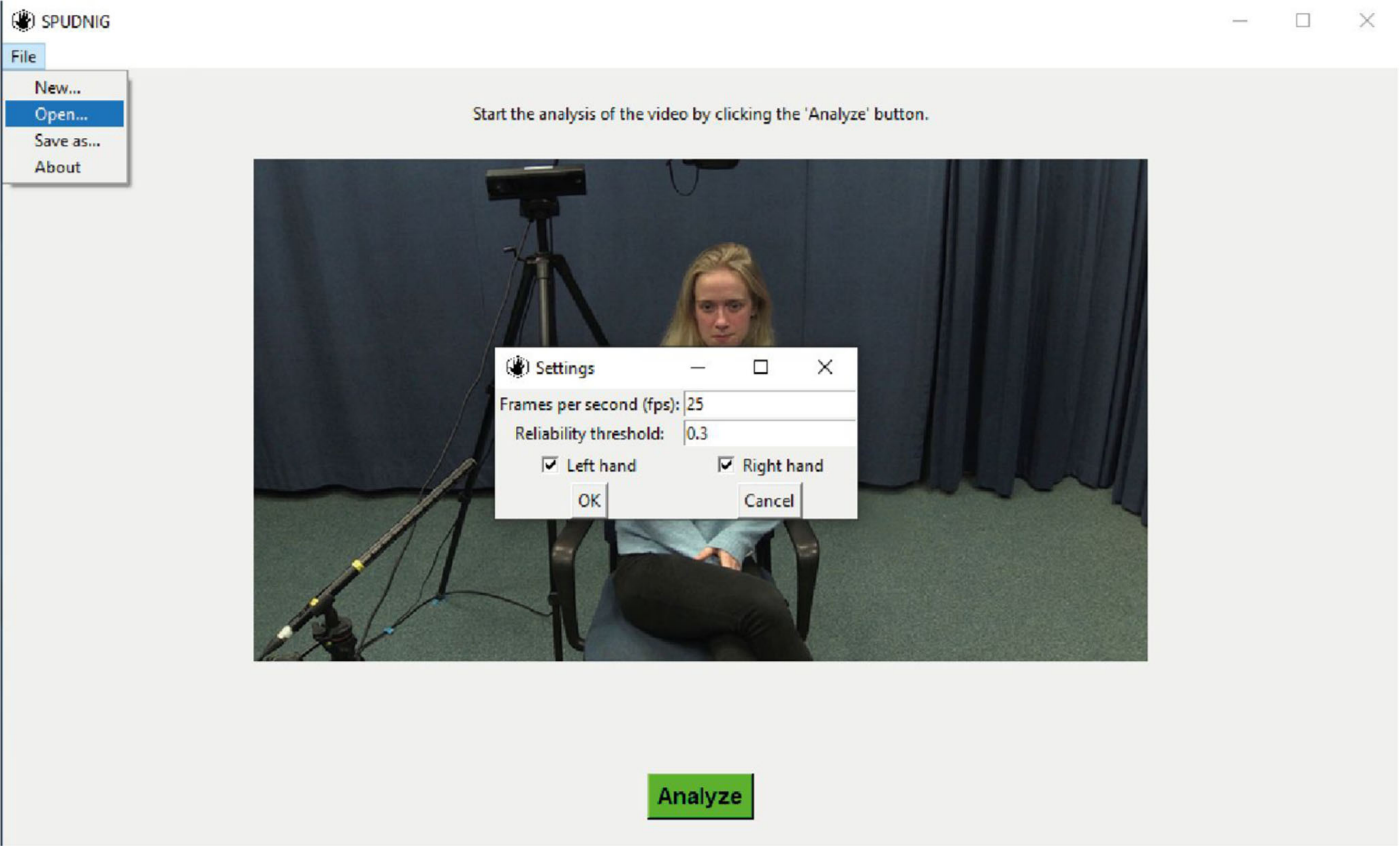
SPUDNIG's start screen. The user can select and load a file by clicking 'File --> Open'. The 'Analyze' button will turn green when a file has been loaded in the application. After clicking the green 'Analyze' button, the user will be prompted by a 'Settings' screen. The default settings are already filled out

The ‘Analyze’ button at the bottom of the screen will turn to green, indicating that the video is ready to be analyzed (see Fig. 2). Pressing the green ‘Analyze’ button will display a parameter selection window (see Fig. 2). After pressing the ‘Analyze’ button, a ‘Settings’ screen appears (see Fig. 2). Here, the frames per second of the video need to be defined (default: 25), as well as the desired reliability threshold (default: 0.3, see above for explanation), and whether the left, right or both hands will be analyzed.

##### Step 3 – Launching analysis and exporting the data

**Fig. 3:**
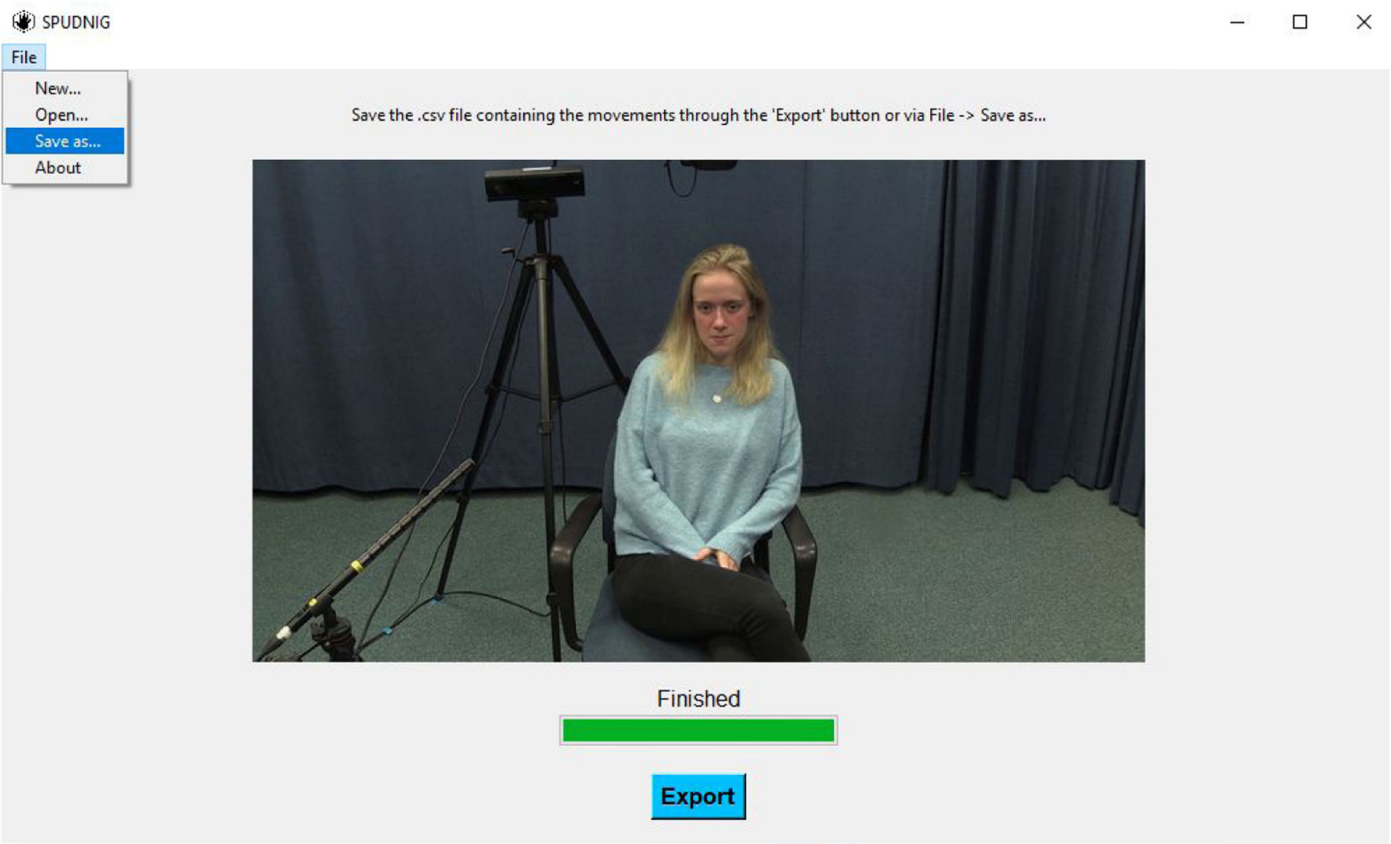
Final screen after the video is analyzed. Users can follow the progress of the analysis in the green bar, and export the resulting .csv file by pressing the blue button

After specifying the desired settings, pressing the green ‘Analyze’ button again will initiate the analysis. Progress of the analysis can be monitored through the green bar, which will fill up during the analysis (see Fig. 3). When finished, the resulting .csv file can be exported by pressing the blue ‘Export’ button, which prompts a window in which the desired save location can be selected (see Fig. 3).

#### Usage notes

1. In addition to our GUI, the underlying source code of SPUDNIG is available on https://github.com/jorrip/SPUDNIG. The parameters described above can, if the user desires, be altered in the source code. Currently, implementation of these features in the GUI is underway (e.g., selection of key points). Additionally, we are planning to add a function that outputs velocity plots per movement (following existing methods, such as for Kinect data, as described by Trujillo et al., 2019).
2. It should be noted that OpenPose performs best when .avi files are used. Other, more complex formats, such as mpeg, might cause OpenPose to skip some frames at the start of videos. This will perturb the timing of the frames and output x/y-coordinates and thus the timing of the detected gestures. Therefore, SPUDNIG currently only takes .avi files as input. Alternative formats can be converted by using free video converters (see our GitHub page for suggested links).
3. Although a GPU is not needed to run SPUDNIG, it can speed up its analysis time. We therefore have a GPU compatible version of our GUI that is available on our Open Science Framework (OSF) repository.
4. Currently, SPUDNIG is designed to only output gestures from a single individual. However, this could easily be extended to analyses of multiple individuals that are visible in a video, as OpenPose can track multiple people.

## Validation analyses

We tested and validated SPUDNIG by comparing its automated annotations of hand movements to manual annotations by a trained human coder. The focus of these analyses was on the detection of gestures. Second, we examined how accurately SPUDNIG could detect both non-iconic and iconic gestures. Finally, we tested whether and how much SPUDNIG accelerates the annotation process by comparing the time it takes to manually annotate videos versus how long it takes to remove false positives that are created by SPUDNIG and adjust, where necessary, the timing of the annotations that are generated by SPUDNIG.

### Corpus

**Fig. 4:**
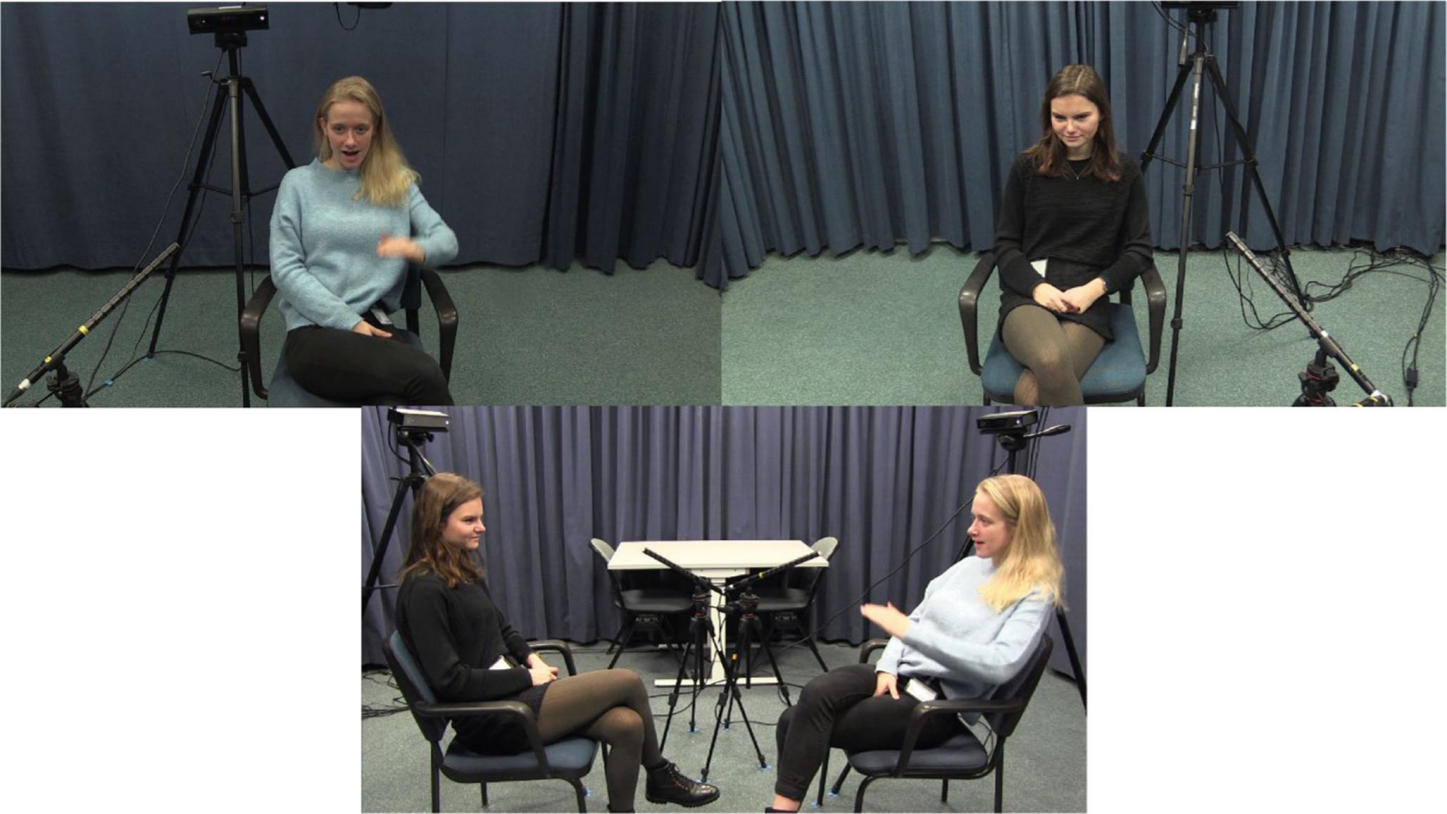
*Upper panels*: Frontal camera view used for data analysis in the SPUDNIG validation test. *Lower panel*: overview of set-up and distance between participants in the corpus used

As a test case for our validation analyses, we used 20 videos that form part of a multimodal communication corpus (CoAct corpus, ERC project #773079). All videos consisted of two acquainted native Dutch speakers that were engaged in dyadic, casual conversations. Participants were recorded while they were having a 1-h conversation. Out of these 1-h conversations, we randomly selected three, 2-min segments per video. These 2-min segments were used to (1) comparing SPUDNIG’s automated annotations of hand movements to manual annotations by a trained human coder to determine how many gestures are detected by SPUDNIG, (2) to test whether SPUDNIG indeed speeds up the annotation process, by comparing how much time untrained and trained human coders take to manually annotate data compared to adjust annotations or remove annotations by SPUDNIG (see below for more detail). All participants were filmed from a frontal perspective while seated on a chair (see Fig. 4).

**Fig. 5:**
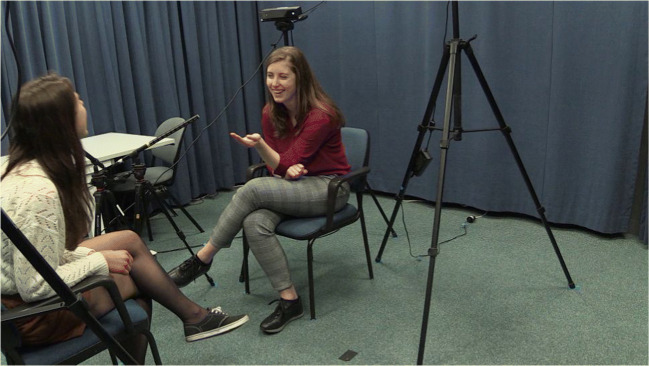
Camera view used while designing and optimizing SPUDNIG. Note that here the speaker’s hands and arms are less visible than from a frontal perspective, and therefore overlap with a human coder might be lower, as some of the key points might be less visible. This might result in less accurate movement detection

Note that SPUDNIG was not created by using these frontal videos, that is, we did not use these frontal videos to optimize our detection algorithm. Instead, we used other videos from the same video corpus that included recordings of the speakers seated at a ~ 45° angle (see Fig. 5). Note that the frontal angle in the videos used for our validation analyses thus provides a more optimal perspective for gesture detection, as the reliability of the key points are likely to be higher in this orientation, and movements can more easily be detected. Overlap with annotations by our human coder might therefore be higher.

### Gesture annotation

We first compared the automated annotations that SPUDNIG made in these videos to manual annotations that were made by a trained, human coder that was blind to the purpose of the coding exercise. This coder was asked to manually annotate the occurrence of gestural movements in the videos, and was asked to include all manual movements that carried some form of meaning. This included movements such as deictic gestures, iconic and metaphoric gestures (depicting aspects of actions, objects space, or abstract concepts), pragmatic gestures (including beats), and interactive gestures (e.g., a palm-up, open-hand gesture that relates to the addressee) (Bavelas, Chovil, Coates, & Roe, 1995; Bavelas, Chovil, Lawrie, & Wade, 1992; McNeill, 1992). The annotations our coder made did not differentiate between these gesture types.

We asked our human coder to annotate all gesture strokes (Kita, van Gijn, & van der Hulst, 1998), including holds and superimposed beats, and to define the begin point of a gesture as the first frame of when the hand left its rest state and its end point as the last frame of the gesture retraction after which the hand remained in rest state (or in the case of successive gestures without retractions, the last frame of the gesture stroke).

Gestures were further annotated in two different ways, one including form-based coding, and one including meaning-based coding. In the form-based coding, every stroke of a gesture was counted as a separate gesture annotation, regardless of whether repeated strokes depicted the same semantic meaning they were part of the same semantic gesture or not (thus, a swimming gesture with multiple successive strokes depicting someone swimming would result in multiple stroke annotations, for example). In the meaning-based coding, individual strokes are combined into one gesture annotation if they clearly depict the same semantic concept and are carried out without perceptible pauses or changes in form (in this case, repeated strokes depicting someone swimming would be annotated as one gesture, for example). The rationale was that both coding approaches seem to be used by gesture researchers and we aimed to make our machine-human coder comparison applicable to both.

For all gestures that were coded in the meaning-based coding tier, we also determined whether the gesture was iconic or non-iconic. This was done to test whether iconic gestures might be better or more easily detected by SPUDNIG than non-iconic gestures. For example, iconic gestures might include fewer holds, which would result in easier detection (because holds cannot be distinguished from non-gesture-embedded rest states by SPUDNIG), or iconic gestures might be larger in size or space, which would result in more clear x/y-coordinate changes than for non-iconic gestures.

### SPUDNIG-human coder reliability analyses

Because SPUDNIG annotates all hand movements in the videos and not just gestures, the question is how this compares to a human coding for gestures. To establish this, we calculated the overlap between all hand movements that SPUDNIG detected and all gestures that were detected by our human coder, for both form-based and meaning-based annotations, as well as distinguishing between iconic gesture and non-iconic gesture annotations for the latter. We calculated a modified Cohen’s kappa between SPUDNIG and our human coder by using EasyDIAg (Holle & Rein, 2015), a standard method for establishing reliability between two human coders. EasyDIAg calculates a modified Cohen’s kappa by taking into account the temporal overlap of the annotations, the categorization of values, and the segmentation of behavior (e.g., when an annotation starts and ends). As recommended, we used an overlap criterion of 60%, indicating that there should be a temporal overlap of 60% between events (i.e., indicating the overlap in movements detected by SPUDNIG and gestures detected by a human).

### Gesture detection accuracy

In addition to trying to identify how many of the gestures the human identified would also be captured by the machine’s movement annotations, we also aimed to identify how much movement detected by the machine was not gestural. Thus, as a second step, we manually compared all raw output from SPUDNIG (i.e., annotations containing all movements in the video) to the output from our human coder to investigate how many movements that were detected by SPUDNIG did not overlap with a gesture annotation. This approach indicated how much of SPUDNIG’s output would have to be filtered out to obtain annotations that solely overlap with gestures, and also indicated how many gestures were not recognized by SPUDNIG, but were recognized by our human coder.

### Does SPUDNIG accelerate the annotation process?

As SPUDNIG annotates all hand movements in the videos and not just gestures, we asked four additional human coders to compare the time it takes to manually annotate the data compared to checking SPUDNIG’s output for whether something is indeed a gesture, and if so, whether the onset and offset of SPUDNIG’s annotation needed to be altered. The human coders were presented with forty, 2-min snippets of video data. Out of these, they were asked to annotate 20 videos manually (i.e., not relying on any pre-annotated data). For the remaining 20 videos, the human coders were asked to use the SPUDNIG output as a basis. A crucial element of this is that SPUDNIG’s algorithm and set threshold result in a highly reliable detection rate of all movements that may constitute gestures. This means that human coders can by-pass the step of checking all frames of a video for possible gestural movement. Instead, with the SPUDNIG output, coders can jump to the already annotated parts and check them for accuracy and then remove false positives and adjust potentially misaligned onsets and offsets.

## Results

### Form-based annotations of gestures

We first compared the overlap between SPUDNIG’s movement annotations and our human coder’s annotations to the form-based gesture annotations by our human coder, and observed 87% raw agreement and a modified Cohen’s kappa maximum value of .86, indicating very high agreement (Cohen, 1960; Landis & Koch, 1977).

Our manual analysis revealed that there were 207 gestures that were identified by our human coder, and 206 by SPUDNIG. Note however that this analysis does not take the amount of overlap between SPUDNIG and the human coder into account, whereas the modified Cohen’s Kappa value does (Holle & Rein, 2015).

### Meaning-based annotations of gestures

We then compared the overlap between SPUDNIG’s movement annotations and our human coder’s annotations to the meaning-based gesture annotations by our coder. We observed 86% raw agreement, and a modified Cohen’s kappa maximum value of .77, indicating a high level of agreement (Cohen, 1960; Landis & Koch, 1977).

Our manual analysis revealed that out of the 185 gestures that were detected by our human coder, 184 were identified by SPUDNIG.

### Iconic gestures

As a next step, we investigated how many of the meaning-based annotations of gestures were iconic gestures. Out of 185 gestures, 45 gestures were iconic. We then compared the overlap between SPUDNIG’s movement annotations and the iconic gesture annotations. Here, we observed 93% raw agreement, and a modified Cohen’s kappa maximum value of 1, indicating almost perfect agreement (Cohen, 1960; Landis & Koch, 1977).

### Non-iconic gestures

Out of the 185 meaning-based annotations of gestures, 140 gestures were non-iconic gestures. We compared the overlap between SPUDNIG’s movement annotations and the non-iconic gesture annotations to the non-iconic gesture annotations. We observed 84% raw agreement, and a modified Cohen’s kappa maximum value of .74, indicating a high level of agreement (Cohen, 1960; Landis & Koch, 1977).

### Movement detection/gesture detection

While SPUDNIG seems to detect movements that constitute gesture highly reliably, it cannot currently recognize gestural from non-gestural movements, leading to considerably more annotations than those that result from a human coding for gestures: SPUDNIG annotated 311 hand movements in the videos, of which 217 were not part of a gesture (= 70%).

### SPUDNIG accelerates the manual annotation process

The crucial test of whether SPUDNIG accelerates the manual annotation process for gestures is based on this high detection rate of movements that potentially constitute gestures. This prerequisite means that our human coders could by-pass the step of checking the videos for false negatives (i.e., any movements SPUDNIG may have overlooked). Instead, they could focus on all extant SPUDNIG annotations to check these for accuracy. Doing so, and correcting the output by removing false positives and adjusting movement onsets and offsets where necessary (in order to make them correspond to gesture on and offsets, or the on and offsets of gesture sequences), significantly sped up our human coders: On average, they were almost twice as quick when using SPUDNIG (mean = 19.3, SD = 12.6, median = 17.8, IQR = 18.4) as compared to manually annotating the data (mean = 35.4, SD = 25.9, median = 33.2, IQR = 24.3), *W* = 235, *p =* 0.02.^1^

## Discussion

We presented SPUDNIG: SPeeding Up the Detection of Non-iconic and Iconic Gestures, a toolkit for the automatic detection of hand movements and gestures in video data. We provided proof-of-principle of our toolkit, and introduced an easy-to-use graphical user interface that researchers can use to automatically annotate hand movements and gestures in their video data.

To validate our method, we used video data containing natural dyadic conversations, and compared SPUDNIG’s output to form-based and meaning-based annotations of gestures, as identified by a trained, human coder, as well as iconic and non-iconic annotations of gestures. The results of our validation analyses demonstrated that our toolkit can very accurately annotate the occurrence of movements that match onto gestures in video data (> 99%) (compared to human generated based on both form and meaning), and irrespective of whether the gesture is iconic or non-iconic. We also note that SPUDNIG leads to a ‘surplus’ of annotations (based on non-gestural movements), meaning that if one is not only interested in annotating human movement per se but in identifying communicative gestures, a human coder is still needed for removing these false positives. However, we have demonstrated that SPUDNIG advances current methods considerably by speeding up the detection of movement that constitutes gestures, the most laborious part of the process, requiring a human coder only primarily for the removal of false positives. Removing such ‘surplus’ annotations is a comparatively much easier and faster process than identifying gestures in a video from scratch. Below we will discuss the performance of SPUDNIG, its implications, and limitations.

### Performance

For all types of gesture coding, SPUDNIG achieved high to very high raw overlap and good to very good modified Cohen’s kappa values (Cohen, 1960; Holle & Rein, 2015; Landis & Koch, 1977). When comparing its performance for iconic and non-iconic gestures, we observed higher raw overlap for iconic gestures than for non-iconic gestures. A possible explanation for this is that iconic gestures might be larger in space or size, and therefore have more easily detectable changes in x/y-coordinates than non-iconic gestures. Non-iconic gestures, for example, might be closer to the threshold for x/y-coordinate changes, and might therefore have less temporal overlap with the annotations of our human coder.

In this regard, it should be noted that SPUDNIG detects all hand movements in the videos, and not only gestures (similar to other semi-automatic gesture detection algorithms, such as Beugher et al., 2018). This means that many of the movements that are part of SPUDNIG’s output need to be manually removed. In all 20 videos that were used for our validation analyses, a total of 311 movements were annotated by SPUDNIG, of which 217 were not part of a gesture (= 70%). These annotations include non-gestural hand and arm movements, such as fidgeting and self-grooming. This percentage is expected to be higher in videos containing natural conversations than in more stimulus-induced or task-based multimodal behavior. Although a substantial percentage of annotations thus needs to be removed by the user, SPUDNIG demonstrated very high gesture annotation overlap, meaning it can be used to save time to identify gestures. By giving the human coder highly reliable annotations of movements that are potentially gestures it can significantly speed up the video data annotation process. This was confirmed in our validation analyses that compared the time it takes human coders to manually annotate a dataset from scratch compared to removing and altering annotations made by SPUDNIG.

### Which gesture did SPUDNIG not detect, and why?

**Fig. 6:**
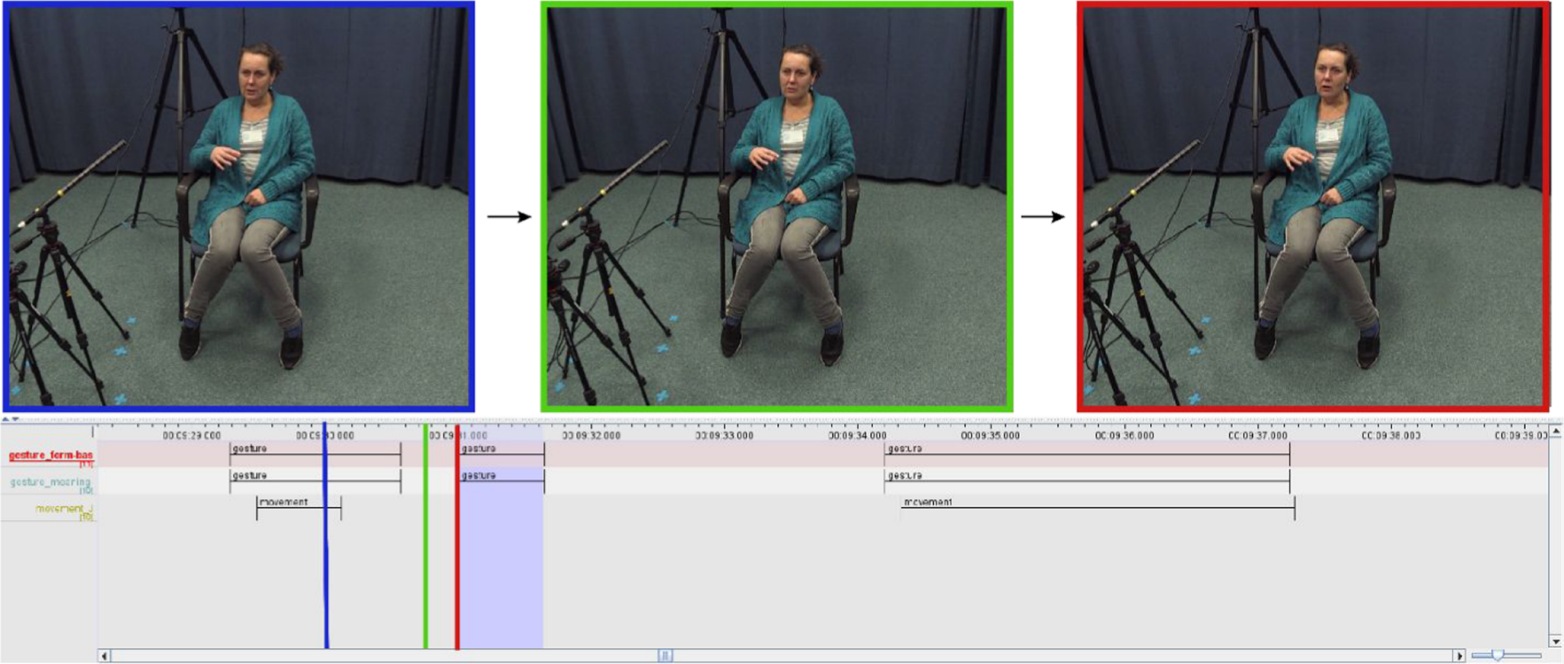
*Upper three panels*: video stills at different moments in time. On the lower panel, the *three vertical colored lines* correspond to the colored squares around the video. The top two tiers include manual annotations from our human coder, indicating the occurrence of gestures. The lowest tier represents movements recognized by SPUDNIG. The *light-purple shaded area* covering the tiers represents the gesture that SPUDNIG missed

In general, 185 gestures (meaning-based coding, 207 in form-based coding) were detected by our human coder, and 184 gestures (meaning-based coding, 206 in form-based coding) were detected by SPUDNIG. One non-iconic gesture was not annotated by SPUDNIG, but was annotated by our human coder (see Fig. 6). As can be observed from the three video stills at different moments in time, the missed gesture remains in approximately the same position from the moment that SPUDNIG detects a movement to the moment that our human coder detects a second gesture. Closer inspection of this gesture, both in the generated .csv files by SPUDNIG and by looking at the video data, revealed that the x/y-coordinates of the key points do not differ much over the span of the two identified gestures. Most probably, SPUDNIG therefore does not detect the second gesture as a separate event, as it consists of a very small change in x/y-coordinates.

Importantly, this example illustrates that SPUDNIG might not be optimal for detecting holds in gestures. In the above-mentioned video, the speaker holds her hand in the same position for a prolonged time. This will be recognized as a rest state by SPUDNIG, and would only be recognized as movement when the pixel change threshold would be lowered. This, on the other hand, would result in an increase of false positives.

Second, SPUDNIG is not optimized for segmenting gestures, as it merges annotations that are separated by four or less frames, and it removes movements shorter than four frames to reduce false positives and negatives. However, this threshold can, if desired, be altered in the source code.

Third, Fig. 6 also shows that the timing of some annotations might need to be adjusted to match the full length of the gesture. As SPUDNIG does not include small movements (i.e., very small changes in x-y coordinates) or very short movements, the onset and offset of annotations might need adjustments by a human coder.

### Implications

In addition to SPUDNIG’s annotations capturing gestures annotated by a human extremely well, SPUDNIG’s main strengths are that using the tool requires no knowledge of programming, no use of motion capture systems, no GPU hardware, and that it comes with an easy-to-use GUI. This makes this tool highly accessible for a large community of users. Moreover, it has the potential to be developed further (based on open source code), with the ultimate aim being a tool that can distinguish gestural from non-gestural movements. While this still requires a big computational leap, the current state of the tool provides the non-programming user with a possibility to significantly speed up current gesture annotation practices.

Finally, the community of users could also be extended to researchers that investigate sign language. As SPUDNIG uses OpenPose as input for its analyses, it would be possible to study finer-grained hand movements and finger movements. These finer-grained movements are usually not detectable by other (semi-)automatic gesture detection systems that use other computer-vision methods (see for a discussion: Beugher et al., 2018). Moreover, SPUDNIG does not require pre-defined regions of interest to look for resting positions or movement. We investigated whether adding user-defined areas of interest for detecting rest states or movements improved detection performance, but we observed that this increased the chance of false negatives.

### Limitations

Although SPUDNIG is highly accurate in detecting hand movements and gestures, it cannot *recognize* different gesture types or forms. Future work could use SPUDNIG’s open source code to add functionalities to recognize gestures. A good starting point would for example be to train SPUDNIG on recurrent gestures (see, for example, Bressem & Müller, 2014, 2017; Ladewig, 2011; Müller, 2017), by which it recognizes certain regular changes in x/y-coordinates over time. However, the recognition of gestures lies beyond the scope of the current manuscript and toolkit.

Second, SPUDNIG’s performance highly depends on how well OpenPose performs on the video data. If the (hands of the) speaker in videos are often occluded (e.g., the speaker sits with their arms folded over each other, or sits on their hands), or the video quality is too low, or the recording angle extreme, it might be difficult to find enough reliable data points to recognize movements. To some extent, these factors can be addressed by altering the threshold for movement detection, but the user should be aware that this has implications for the false positives and negatives in the output.

Third, SPUDNIG can be used on 2D data, but does not provide information on movements in three-dimensional space. It therefore seems less suited to study complex movement dynamics related to directionality or space (see for example Trujillo et al., 2019, where 3D information is used to study detailed kinematic features).

Finally, SPUDNIG uses pixel coordinates as thresholds for detecting movements. An alternative way to analyze the OpenPose output is by using millimeters as thresholds, which can be achieved by using the camera calibration procedure that is already part of OpenPose. This could result in a more generic threshold when two different cameras capture the same movements, and the resolution of these two cameras differs. This option is currently not supported in SPUDNIG.

## Conclusions

We demonstrated that SPUDNIG highly accurately detects iconic and non-iconic gestures in video data. SPUDNIG aims to accelerate and facilitate the annotation of hand movements in video data, and provides an easy-to-use and quick alternative to the labor-intensive and time-consuming manual annotation of gestures.

## Footnotes

1 As a comparison, we also asked our coders to use the SPUDNIG output (with a different set of 20 video snippets) to check all non-annotated frames for potential false negatives, in addition to making corrections for false positives and on and offsets. With such a procedure, we still see a benefit in terms of average annotation time, but one that is less pronounced (with SPUDNIG output: mean = 76.8 ms, SD = 83.4 vs. manually annotating the data without SPUDNIG output: mean = 81.2 ms, SD = 77.06). This means that, even when coders do not make full use of the tool by mistrusting SPUDNIG to detect potential gestural movements with high accuracy, we observe a benefit in terms of annotation time. However, note that the full benefit results from the tool’s ability to maximize human coders’ efforts by focusing them on to-be-checked pre-annotated segments.
